# Early evolvability in arthropod tagmosis exemplified by a new radiodont from the Burgess Shale

**DOI:** 10.1098/rsos.242122

**Published:** 2025-05-14

**Authors:** Joseph Moysiuk, Jean-Bernard Caron

**Affiliations:** ^1^ The Manitoba Museum, Winnipeg, Manitoba, Canada; ^2^ Geological Sciences, University of Saskatchewan, Saskatoon, Saskatchewan, Canada; ^3^ Natural History-Paleobiology, Royal Ontario Museum, Toronto, Ontario, Canada; ^4^ Ecology and Evolutionary Biology, University of Toronto, Toronto, Ontario, Canada; ^5^ Earth Sciences, University of Toronto, Toronto, Ontario, Canada

**Keywords:** segmentation, convergence, Cambrian explosion, functional specialization, body plan, arthropod

## Abstract

Much diversity in arthropod form is the result of variation in the number and differentiation of segments (tagmosis). Fossil evidence to date has suggested that the earliest-diverging arthropods, the radiodonts, exhibited comparatively limited variability in tagmosis. We present a new radiodont, *Mosura fentoni* n. gen. and n. sp., from the Cambrian (Wuliuan) Burgess Shale that departs from this pattern. *Mosura* exhibits up to 26 trunk segments, the highest number reported for any radiodont, despite being among the smallest known. The head is short, with a small, rounded preocular sclerite, three prominent eyes and appendages with curving endites tipped with paired spines, altogether suggesting a nektonic, macrophagous predatory ecology. The trunk is divided into a neck, mesotrunk with large swimming flaps and multisegmented posterotrunk with tightly spaced bands of gill lamellae and reduced flaps. Detailed preservation of expansive circulatory lacunae, closely associated with the gills, clarifies the nature of similar structures in other Cambrian arthropod fossils, including *Opabinia*. The morphology of the posterotrunk suggests specialization for respiration, unique among radiodonts, but broadly convergent with the xiphosuran opisthosoma, isopod pleon and hexapod abdomen. This reinforces the hypothesis that multiple arthropod lineages underwent parallel diversification in tagmosis, in tandem with their initial Cambrian radiation.

## 1. Introduction

The differentiation of different batches of body segments—tagmosis—is perhaps the most ubiquitous characteristic of arthropods. Specific patterns of tagmosis frequently characterize major arthropod clades. For example, various malacostracan crustacean groups like crabs, isopods and amphipods exhibit particular patterns of tagmosis, with different appendages adapted for sensation, feeding, respiration, walking, swimming, brooding eggs, grooming and more [1]. With some exceptions, tagmosis is frequently a fairly conserved trait within clades, but at broader scales there is evidence that increasing tagmosis has been a pervasive trend in arthropod macroevolution [2–4]. The implication is that a greater number of tagmata may be selectively favourable owing to the capacity of each tagma to become functionally specialized, releasing evolutionary constraint due to trade-offs [5]. On this basis, it is often posited that variability in tagmosis has been a driving factor in arthropod diversification throughout evolutionary history [6].

Although the evidence for an increase in tagmosis over time appears to be clear, the origin, tempo and mode of this trend remains poorly constrained. It was long hypothesized that the ancestral panarthropod had simple tagmosis and that arthropod tagmosis increased gradually over deep time across multiple lineages [2,7]. This notion seems intuitively supported when considering the closest living panarthropod relatives of arthropods, onychophorans and tardigrades, which have few tagmata and essentially invariant patterns of tagmosis, close to the presumed ancestral state [8,9].

Accumulating fossil evidence points to a somewhat different hypothesis: that panarthropods underwent a pulse of early diversification in tagmosis, with relatively less change thereafter in persisting lineages. Indeed, it has become increasingly clear that the extinct lobopodian grade that gave rise to all living panarthropods exhibited greater diversity in tagmosis than extant tardigrades or onychophorans [10,11]. Similarly, varied patterns of tagmosis are now being recognized in Cambrian euarthropod groups such as isoxyids [12,13], hymenocarines [14–18], fuxianhuiids [19,20], pancrustaceans [21,22], panchelicerates [23,24] and artiopodans [25–29]. In this framework, extant clades with relatively constrained tagmosis, from tardigrades and onychophorans to hexapods and decapods, therefore represent canalized remnants of an early and relatively rapid radiation [30].

One clade that has thus far appeared to defy this emerging pattern is Radiodonta. Radiodonts are the earliest diverging arthropods, a stem group to the clade Euarthropoda *sensu* [31]. Given their phylogenetic position, radiodonts provide key evidence as to ancestral morphological, ecological and developmental traits in arthropods (reviewed in [32]). All previously described radiodonts are characterized by a frontal appendage pair specialized for feeding and a trunk region comprising a series of segments bearing flap-like appendages used primarily for locomotion. Invariably, the trunk can be subdivided into three batches of segments: (i) an anterior neck, characterized by three to six segments with smaller flaps [33–37] and possible gnathal elements in some species [38,39]; (ii) a 7- to 14-segmented region in which segment and flap size is generally large at the anterior and decreasing in size gradually towards the posterior [40,41]; and (iii) a posteriormost tagma comprising one to four pairs of blade- or filament-like structures [35,41–43], which are presumed to be appendicular in origin and at least in some cases functioned as rudders [44]. Compared to other groups of Cambrian panarthropods, radiodonts therefore appear to exhibit a modest level and relatively conservative pattern of tagmosis.

Here, we present a new species of radiodont with a divergent pattern of morphofunctional division of trunk segments from the mid-Cambrian (Wuliuan) Burgess Shale. The new species shows that radiodonts also evolved diverse patterns of body regionalization convergently with various euarthropod lineages. In addition, these fossils help to clarify the nature of segmentally arranged, reflective, triangular structures, which have been previously observed in *Opabinia* and a variety of other arthropod fossils of Burgess Shale type.

## 2. Material and methods

Sixty specimens were collected over the course of nine field seasons between 1990 and 2022 from the Burgess Shale Formation, British Columbia, Canada, specifically the Raymond Quarry in Yoho National Park, and various localities around Marble Canyon [45] and Tokumm Creek [46] in Kootenay National Park. These are reposited at the Royal Ontario Museum Invertebrate Palaeontology collections (ROMIP). A single specimen (USNM 275685) was additionally identified in the collections of the Smithsonian National Museum of Natural History (NMNH). Some ROMIP specimens were mechanically prepared using an air scribe with a pointed 2 mm needle (Hardy Winkler Drucklufthammer HW-70) to remove the sediment matrix, coating parts of the fossils. For specimens that were sufficiently complete, sagittal length measurements were taken (see electronic supplementary material, table S1.1 for the full list of specimens).

Specimens were variously photographed under polarized and non-polarized light and submerged in water to bring out different aspects of fossil morphology. Interpretive line drawings of specimens were created by combining information observable under different imaging conditions. Elemental mapping was carried out on one specimen (figure 5) with an environmental scanning electron microscope (FEI Quanta 200) equipped with energy dispersive spectroscopy using an EDAX Octane Plus Silicon Drift X-ray detector at the University of Windsor Great Lakes Institute for Environmental Research, Canada. Element analysis of a second specimen (electronic supplementary material, figure S5) was obtained at the University of Toronto in the Earth Sciences Department using an environmental scanning electron microscope (JEOL JSM 6610LV) equipped with an Oxford X-Max X-ray detector. Both imaging analyses were conducted in high vacuum mode using 10 or 12 kV beam accelerating voltage.

Phylogenetic analysis utilized a comprehensive dataset from [41] with the addition of the new taxon and modifications based on morphological revisions of other taxa from new publications (148 taxa and 322 morphological characters; see electronic supplementary material for details). Bayesian phylogenetic analysis was carried out in MrBayes 3.2.7a (parallel version) [47] using similar parameters to those in [41]. Specifically, based on prior model testing, we employed a Markov *k*-states model with gamma-distributed rate heterogeneity, a correction for sampling only parsimony-informative characters [48] and separate character partitions for neomorphic and transformational characters, allowing asymmetrical transition frequencies in the former (Mki+Γ+NT model [49]). Backbone constraints based on molecular phylogenetic topologies were also included for specific extant taxa. The analysis was set to stop when average standard deviation of split frequencies reached 0.005, with convergence confirmed in Tracer 1.7.2 [50] and by examining the set of trees. We summarized the set of posterior trees with a majority rule consensus as well as calculating the maximum clade credibility tree (MCC) and investigating clade support using monophyly testing (*sensu* [41]) using functions from the R (v. 4.4.0 [51]) packages *ape* [52], *phangorn* [53] and *geiger* [54].

In order to interpret the function of the specialized posterior tagma of the new taxon, we sought to estimate the relative size of the gills compared to other radiodonts. Specimens of *Anomalocaris canadensis* [34], *Innovatiocaris maotianshanensis* [33], *Lyrarapax unguispinus* [37,55], *Stanleycaris hirpex* [41], *Peytoia nathorsti* [56] and *Cambroraster falcatus* [36] were dorsoventrally oriented and sufficiently complete for comparison (electronic supplementary material, table S1.2). Measurements were taken from photographs using ImageJ [57]. Owing to the compression of the fossils and the overlapping of anatomical structures, it was not possible to measure gill surface area or body mass/volume reliably. As a proxy for body size, we measured total body length. As a rough proxy for gill area, we estimated the length of each band of gill lamellae as the width of the trunk at the lengthwise midpoint of each segment. This is likely an underestimate, as the gill bands extend onto the proximal portion of the flaps, but taking this approach allowed consistent measurement of a reasonable sample of specimens. We then summed the individual gill lengths to get a measure of total gill length. The caveat of this approach is that it is not possible to account for differences in relative gill area between segments or taxa, so it must be considered a tentative estimation.

## 3. Systematic palaeontology

urn:lsid:zoobank.org:pub:1F6FFE13-BEF7-41E4-8D79-D0A0DB1488CC

Superphylum Panarthropoda Nielsen 1995 [58]

Phylum Arthropoda Gravenhorst 1843 [59,60]

Order Radiodonta Collins 1996 [43]

Family Hurdiidae Lerosey-Aubril & Pates 2018 [61]

Genus *Mosura* n. gen.

urn:lsid:zoobank.org:act:C9EDD74C-5EC7-40C6-8B9B-E2D2780CC18A

*Type species: M. fentoni* n. sp., by monotypy.

*Etymology*: From the name of the fictional Japanese monster, or kaiju モスラ (also known as ‘Mothra’), romanized according to Hepburn style, in reference to the moth-like appearance of the animal.

*Diagnosis*: As for species, by monotypy.

*Mosura fentoni* n. sp.

urn:lsid:zoobank.org:act:26A8C982-AE59-40C3-8F0B-13F3AE6CD236

*Holotype*: ROMIP 67995, part and counterpart, a complete specimen preserved in dorsal view (figure 2*a*–*d*).

*Etymology*: In honour of Peter E. Fenton, for his 40 years of service as a technician in the Invertebrate Palaeontology section at the Royal Ontario Museum, and for his unwavering friendship to both authors.

*Diagnosis*: Radiodont with an adult trunk region divided into a four-segmented neck, six-segmented mesotrunk and up to 16-segmented posterotrunk. Flaps of mesotrunk up to *ca* 60% of core segment width. Flaps of posterotrunk markedly differentiated in size, less than *ca* 20% of core segment width. Caudal blades absent, posterotrunk terminating in pair of small, triangular processes. Appendages with six elongate endites (*ca* 3.5 times podomere height) with bifurcated tips. Lateral eye stalks short, less than eye diameter.

*Description*: Specimens range from 15 to 61 mm in total body length. Size distributions from the Tokumm Creek and Raymond Quarry sites are largely overlapping; Tokumm specimens tend to be slightly larger, but the difference is not significant (*p* = 0.09; electronic supplementary material, figure S1). We find no other qualitative differences between these populations.

**Figure 1.**
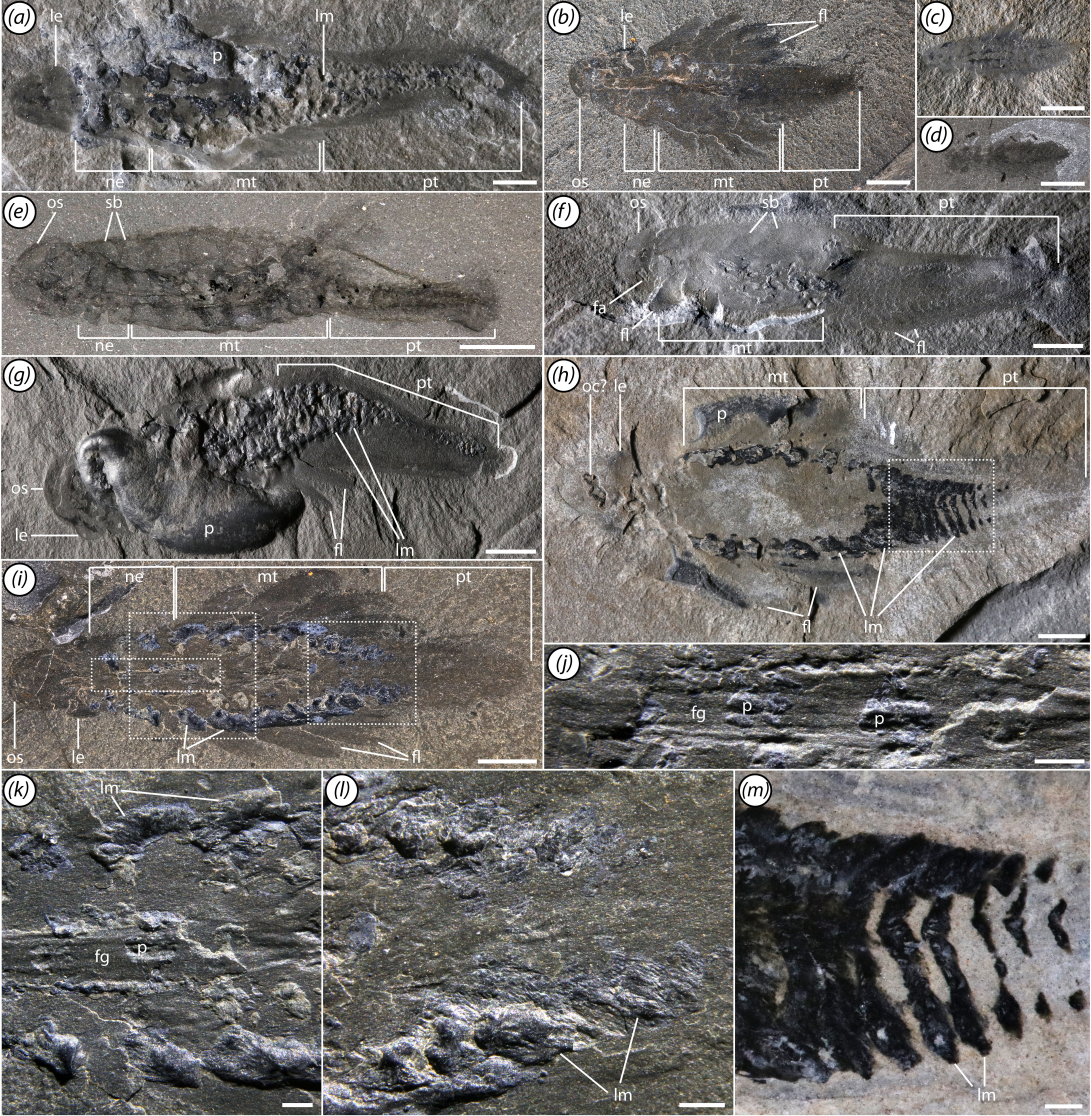
General overview of morphology. (*a*) ROMIP 67520, shown at same scale as panels (*b–d*) to illustrate size variation; (*b*) ROMIP 67983; (*c*) ROMIP 67979; (*d*) ROMIP 68000; (*e*) ROMIP 68006, lateral view; (*f*) ROMIP 67060, lateral view; (*g*) ROMIP 67058; (*h*) ROMIP 67974; (*i–l*) ROMIP 67993. (*i*) Specimen overview, (*j–l*) close-ups from (*i*): phosphatic elements along the foregut and phosphatized lamellae (*j,k*), and phosphatized lamellae along the posterior trunk; (*m*) close-up of part of the posterior trunk from (*h*). Scale bars, (*a–i*) 5 mm; (*j–m*) 1 mm. Abbreviations: fa, frontal appendage; fg, foregut; fl, flap; le, lateral eye; lm, band of lamellae; mt, mesotrunk; ne, neck; oc, oral cone; os, preocular sclerite; p, phosphatized element; pt, posterotrunk; sb, segmental boundary.

The body can be broken down into four regions: a head, neck, mesotrunk and posterotrunk (figure 1*a*–*i*). The last three consist of multiple segments bearing flaps and bands of lamellae. The flaps project ventrolaterally from the body (figure 1*f*). Dorsally, the body is strongly convex and shows evidence of distinct segmental boundaries (figure 1*e,f*).

**Figure 2.**
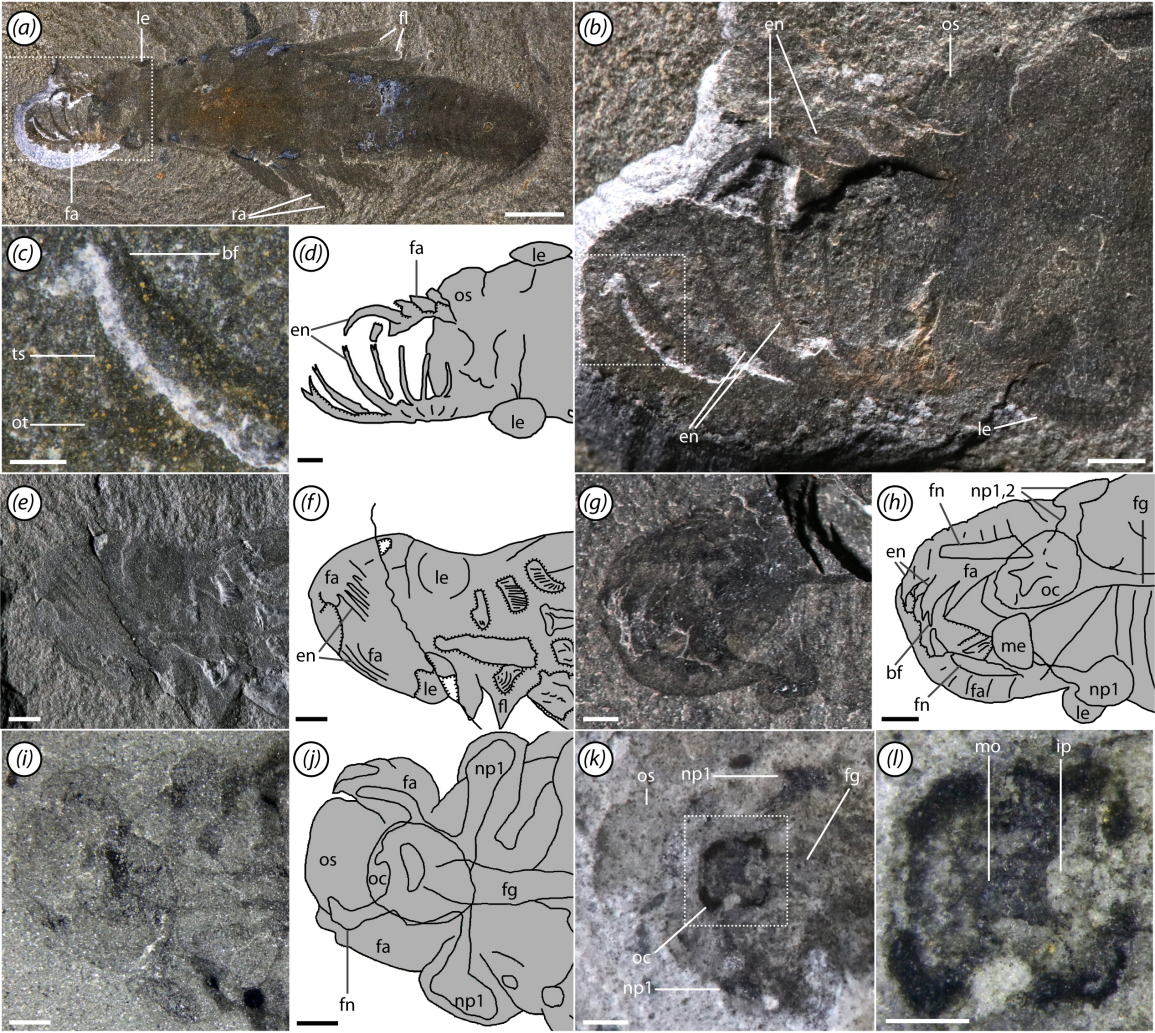
Appendages and oral cone. (*a–d*) ROMIP 67995 (holotype), ventral view, (*a*) overall specimen, (*b*) close-up of head, (*c*) close-up of one endite from frontal appendage, (*d*) interpretive line drawing; (*e,f*) ROMIP 68005, (*e*) ventral view of head, (*f*) interpretive line drawing; (*g,h*) ROMIP 66108, (*g*) ventral-oblique view of head, (*h*) interpretive line drawing, combining information from alternative lighting conditions (figure 4*a*); (*i,j*) ROMIP 68004, (*i*) dorsal view of head, (*j*) interpretive line drawing; (*k,l*) ROMIP 67982, (*k*) close-up of head, (*l*) close-up of oral cone. Scale bars, (*a*) 5 mm; (*b,d–k*), 1 mm, (*c,l*) 0.5 mm. Abbreviations: bf, bifurcated tip of endite; en, endite; fa, frontal appendage; fg, foregut; ip, inner plates of oral cone; le, lateral eye; me, median eye; mo, mouth; np#, lateral eye neuropil #; oc, oral cone; os, preocular sclerite; ot, outer spine of appendage; ra, flap ray; ts, terminal spine of appendage.

**Figure 3.**
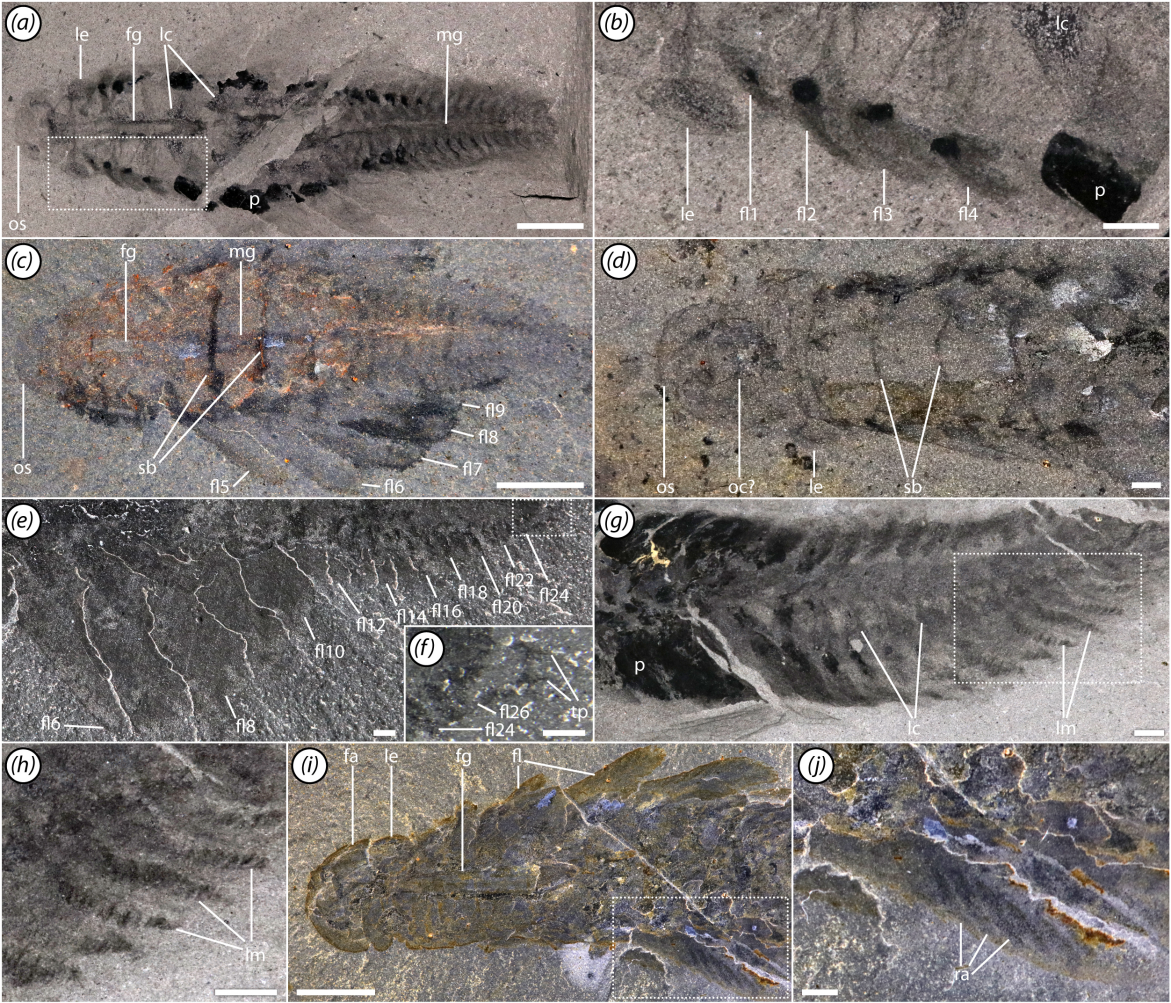
Lateral flaps and gills. (*a,b*) ROMIP 68004, (*a*) overall specimen, (*b*) close-up of nuchal flaps; (*c*) ROMIP 67983, overall specimen; (*d*) ROMIP 41466, close-up of head and neck regions; (*e,f*) ROMIP 66108, (*e*) lateral side of body showing flaps, (*f*) close-up of posterior termination; (*g,h*) ROMIP 68012, (*g*) view of posterotrunk, (*h*) close-up of several gills showing lamellae; (*i,j*) ROMIP 68007, (*i*) front half of specimen, (*j*) close-up of flaps showing structural rays. Scale bars, (*a,c,i*) 5 mm; (*b,d,e–h,j*) 1 mm. Abbreviations: fa, frontal appendage; fg, foregut; fl, flap; lc, lacuna of circulatory system; le, lateral eye; lm, band of lamellae; mg, midgut; os, preocular sclerite; p, phosphatized element; ra, flap ray; sb, segmental boundary; tp, terminal process.

The head is an externally undivided structure emitting the eyes and frontal appendages, as well as the oral cone and preocular sclerite. The head is roughly subrectangular in dorsal view and relatively short, taking up *ca* 15% of total body length (figure 1*a*–*i*). The preocular sclerite is approximately as wide as the head and semicircular (figures 1*b*,*e*–*g*,*i*, 2*a*,*i*–*k* and 3*a*,*c*,*d*; electronic supplementary material, figure S2). Only the outline of the oral cone is typically visible, taking up *ca* 40% head width (figure 2*g*–*l*; electronic supplementary material, figures S2 and S3). In one specimen, the oral cone appears as a dark quadrate outline surrounding four semicircular lighter regions that flank a darker central region (figure 2*k,l*; electronic supplementary material, figure S4). This suggests a tetraradial plate organization, although individual plates cannot be discerned. Owing to their shape and position, we interpret the semicircular regions as inner oral plates, similar to those seen in *Hurdia* [62] and *Cambroraster* [36]. The darker, partially phosphatized, quadrate rim associated with the oral cone recalls similar structures in some specimens of *Lyrarapax* [37] and *Stanleycaris* [63, fig. 3*c*], potentially representing the remains of internal soft tissues [55].

**Figure 4.**
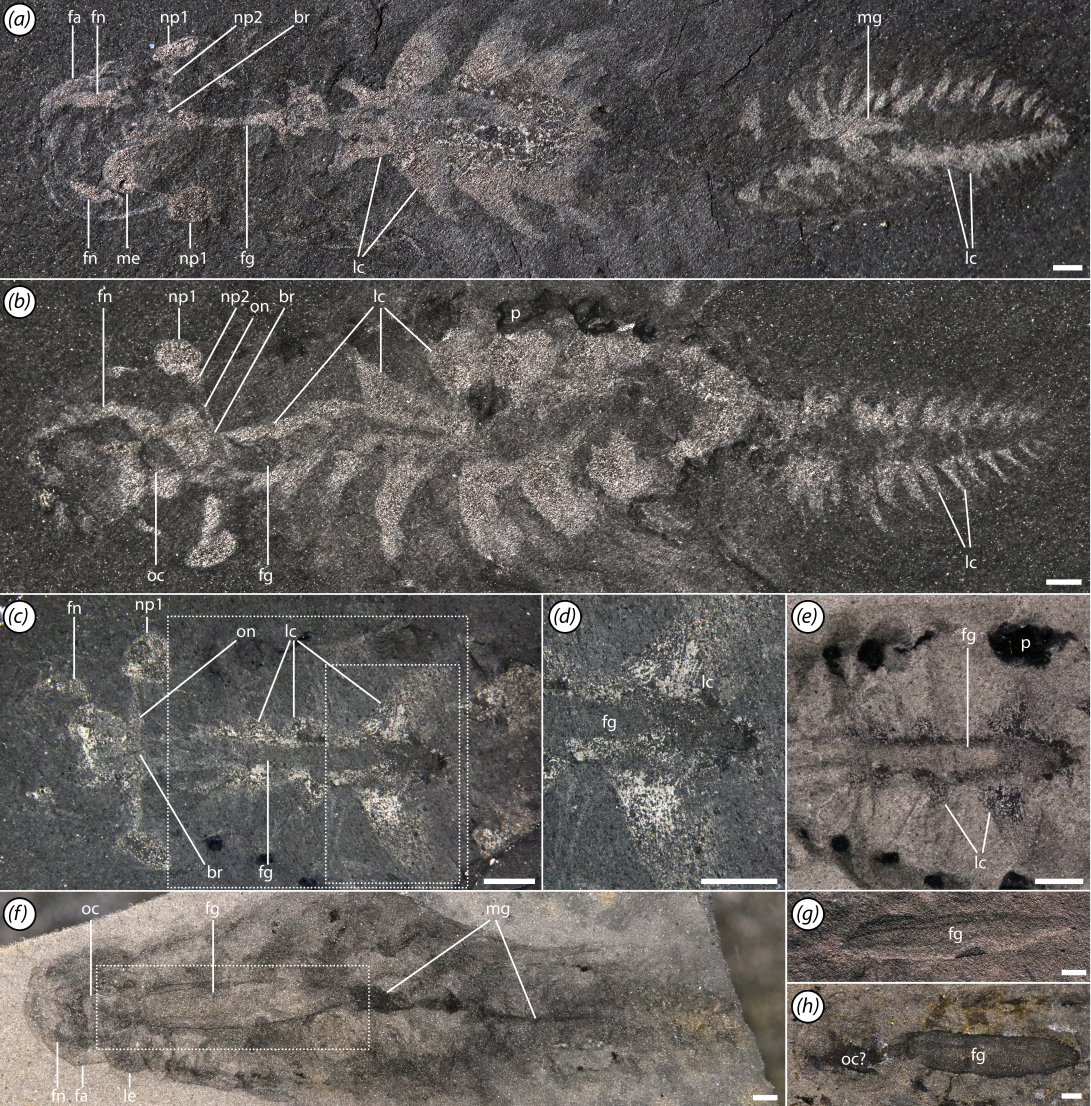
Internal organ systems. (*a*) ROMIP 66108, overall specimen, lit with high angle light and submerged in water; (*b*) ROMIP 67998, overall specimen, lit with high angle light and submerged in water; (*c–e*) ROMIP 68004, (*c*) front half of specimen, lit with high angle light and submerged in water, (*d*) close-up of triangular trunk lacunae showing striated structure, possibly associated with musculature, (*e*) neck region, under cross polarized lighting; (*f,g*) ROMIP 67999, (*f*) overall specimen, under cross polarized lighting, (*g*) close-up of foregut, high angle lighting; (*h*) ROMIP 41466, view of head and foregut. Scale bars, 1 mm. Abbrevations: br, brain; fa, frontal appendage; fn, frontal appendage nerve/circulatory lacuna; fg, foregut; lc, lacuna of circulatory system; le, lateral eye; mg, midgut; np#, lateral eye neuropil #; oc, oral cone; on, optic nerve; p, phosphatized element.

**Figure 5.**
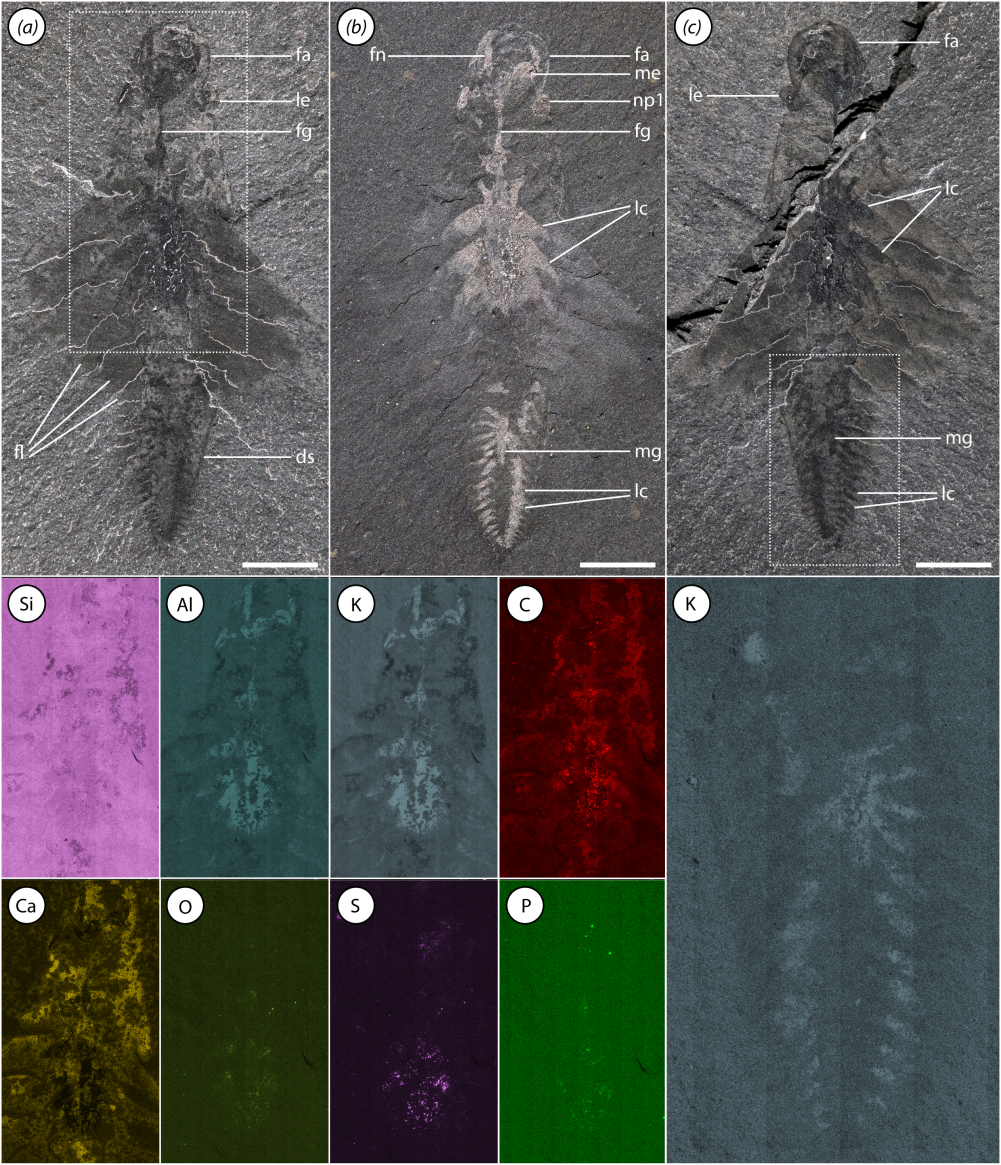
Additional views of ROMIP 66108, including elemental maps. (*a*) Part under polarized light; (*b*) part under high angle light; (*c*) counterpart under polarized light. Lower panels are close-ups of anterior (left) and posterior (right) regions of body showing the distribution of indicated elements with enrichment proportional to brightness. Scale bars, 5 mm. Abbreviations: ds, dorsal surface of body; fa, frontal appendage; fg, foregut; fl, flap; lc, lacuna of circulatory system; le, lateral eye; me, median eye; mg, midgut; np#, lateral eye neuropil #.

The appendages are only observable in a few specimens, as they are usually tucked beneath the head and impressed against other tissues (figure 2*e*–*h*). A row of six elongate, curving endites are present towards the proximal end of the appendage, each connecting to a single podomere (figure 2*a*–*d*). The endites range from three to four times the height of the supporting podomeres. Each endite is sickle-like in shape and tipped with a pair of small spines (figure 2*c*). Additional auxiliary spines are absent. Although no podomere boundaries are preserved proximal to the proximalmost endite, the attachment position of the latter suggests that an additional podomere, the peduncle, is likely present. Similarly, distal to the elongate endites, the number of podomeres cannot be counted, but based on the length of this section of the appendage, we would estimate that at least three were likely present. The appendage ends with a pair of stout, gently curving spines (figure 2*b*–*d*). We tentatively interpret the inner of these two as a terminal spine and the outer one as equivalent to the outer spines seen in other radiodonts [64]. No other cuticular outgrowths can be observed. Internally, reflective traces extend through the appendages, probably representing internal tissues including nerves and surrounding lacunae (figures 2*i*,*j*, 4*a*–*c* and 5; electronic supplementary material, figures S2 and S3; see §5).

A pair of lateral eyes are situated on short peduncles (figures 1*a*–*d*,*g*–*i*, 2*a*,*b*,*d–k*, 3*a*–*d*,*i* and 4*a*–*c*,*f*). Eye diameter is *ca* 40% the length of the head. Reflective internal traces are interpreted as large first optic neuropils (figures 2*g*–*k* and 4*a*–*c*; electronic supplementary material, figures S2 and S3), similar to those observed in other radiodonts [37,41]. These connect to subconical traces narrowing towards the sagittal axis, interpreted as second optic neuropils (figure 4*a*,*b*; electronic supplementary material, figure S3). These are in turn connected via thin optic nerves to a reflective axial patch in the head, which we interpret as remains of the brain and surrounding perineural sinus of the lacunar system (figure 4*a*–*c*; electronic supplementary material, figure S2).

**Figure 6.**
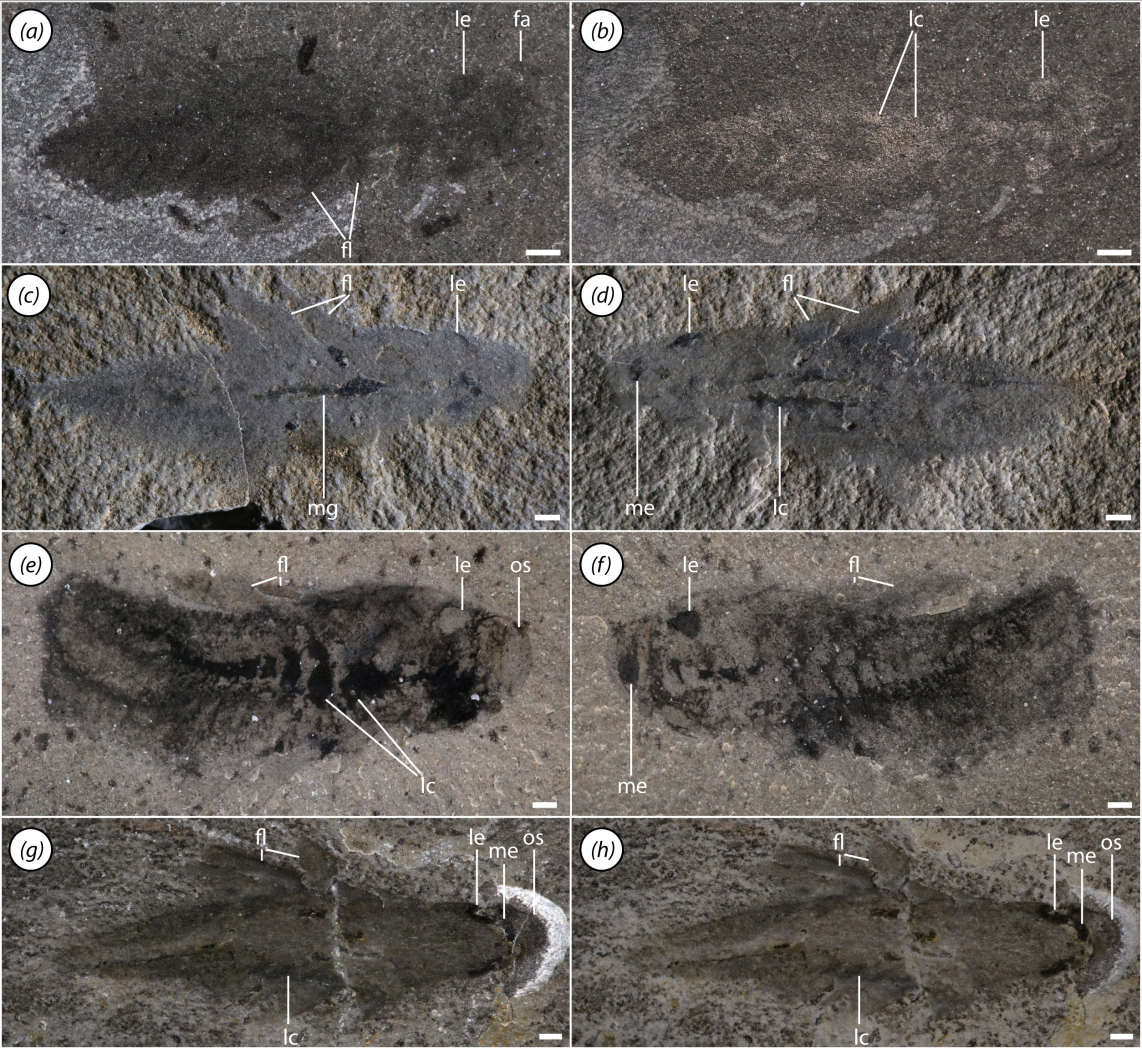
Juvenile specimens of *Mosura fentoni*. (*a,b*) ROMIP 68000; (*a*) polarized light; (*b*) high angle light; (*c,d*) ROMIP 67979, high angle light; (*c*) part; (*d*) counterpart; (*e,f*) ROMIP 67510, polarized light; (*e*) part; (*f*) counterpart; (*g,h*) ROMIP 67083, polarized light; (*g*) dry; (*h*) wet. Scale bars, 1 mm. Abbreviations: fa, frontal appendage; fl, flap; lc, lacuna of circulatory system; le, lateral eye; me, median eye; mg, midgut; os, preocular sclerite.

An elliptical medial structure, reflective under low-angle light and of equivalent elemental composition to the neuropils of the lateral eyes is interpreted as a median eye neuropil (figures 2*g*,*h*, 4*a*, 5 and 6*c*–*h*; electronic supplementary material, figure S4c,d). The median eye is roughly the same size as the lateral eyes. It presumably innervates the anteromedial region of the brain, although the median eye nerve is not clearly distinguishable.

The segments of the trunk are delimited by clear boundaries in the dorsal cuticle (figure 1*e,f*). Each segment also bears a band of lamellae, which is continuous across the sagittal axis and is apparently positioned ventrally (figures 1*a*,*g*–*i*,*k*–*m* and 3*g*,*h*). The lamellae are frequently phosphatized, with individual lamellae occasionally visible (figure 1*a*,*g*–*i*,*k*–*m*), although in rarer cases they may be preserved as carbonaceous traces (figure 3*g*,*h*).

The neck, making up the anterior portion of the trunk region, comprises four flap-bearing segments (figure 3*b*). The flaps are short and the segments become relatively wider towards the posterior.

The mesotrunk comprises six segments bearing elongate flaps (figures 1*b*, 2*c* and 5). Flaps are broad and triangular (figure 3*e*), though they may appear more sausage-shaped when directed posteriorly due to compressional telescoping and imbrication (figure 2*a*). Each flap is transected by a series of thick parallel rays oriented at an anterodistal-to-posteroproximal angle (figures 2*a* and 3*i*,*j*). Probably 12−15 rays are present in the largest flaps. Mesotrunk flap length increases from the first to second pair, then decreases posteriorly. At maximum size, mesotrunk flaps are over 60% of core trunk width. The sixth mesotrunk flaps are relatively small, transitional with those of the succeeding posterotrunk (figure 3*c*,*e*). The smallest *Mosura* specimens (figures 1*c*,*d* and 6), show only three pairs of larger flaps in the mesotrunk, though it is possible that additional flaps were present but not observable.

The posterotrunk consists of up to at least 16 segments in specimens ranging from 35 to 46 mm in length (figure 3*a*,*e*). Due to the small size of posterior segments, segment number in the posterotrunk can be counted precisely in only a few specimens, so it is unclear whether this represents the maximum segment number. The number of segments in the posterotrunk also appears to be lower in the smallest specimens (figure 6: *ca* 8 in figure 6*a*,*b* and *ca* 14 in figure 6*c*,*d*) although the precise number is not possible to count owing to small size. Segments of the posterotrunk are very short and tightly packed, and the associated flaps are highly reduced in size (figure 3*e*). Flaps of the first posterotrunk segment are roughly 20% of the width of their associated segment, and relative flap size decreases posteriorly until they are no longer visible. Posterotrunk segments narrow gradually to the 22nd segment and then narrow rapidly to a rounded termination. A pair of small, triangular terminal processes is present (figure 3*f*).

The foregut is quite elongate and extends up to the fifth trunk segment, being about 30% of maximum trunk width excluding flaps (figures 1*i*–*k*, 2*g–l*, 3*a*,*c*,*i* and 4; electronic supplementary material, figures S2 and S3a–e). It may be preserved in slight three-dimensional (3D) relief, with primarily clay minerals, although some calcium phosphate may be present (figure 1*j,k*). The midgut is simple, tubular and is preserved as a dark, carbon-aluminosilicate trace through most of the trunk region (figures 3*a*,*c*, 4*a*,*f* and 5). It is always incompletely preserved and often shows signs of rupturing when present. No hindgut is evident.

Reflective patches surround the gut and extend as broad triangular tonguelettes into the flaps (figures 4*a*–*e* and 5; electronic supplementary material, figures S2 and S3). Preservation of these structures is quite variable; in some specimens they may fill a large proportion of the body trace, while in others, only their lateral projections into the flaps are visible. In appearance and preservation style, these traces are similar to reflective patches in the head discussed previously. We interpret these as primarily representing remains of internal body cavities of the lacunar system. As with gill lamellae, areas apparently associated with lacunae, particularly near distal tips of the triangular tonguelettes, may be 3D phosphatized (figures 1*a*, 3*a*,*b*,*g* and 4*b*,*e*). Several specimens also exhibit such extreme overgrowth of calcium phosphate that the original tissue being replicated is uncertain (figure 1*g*; electronic supplementary material, figure S5).

**Figure 7.**
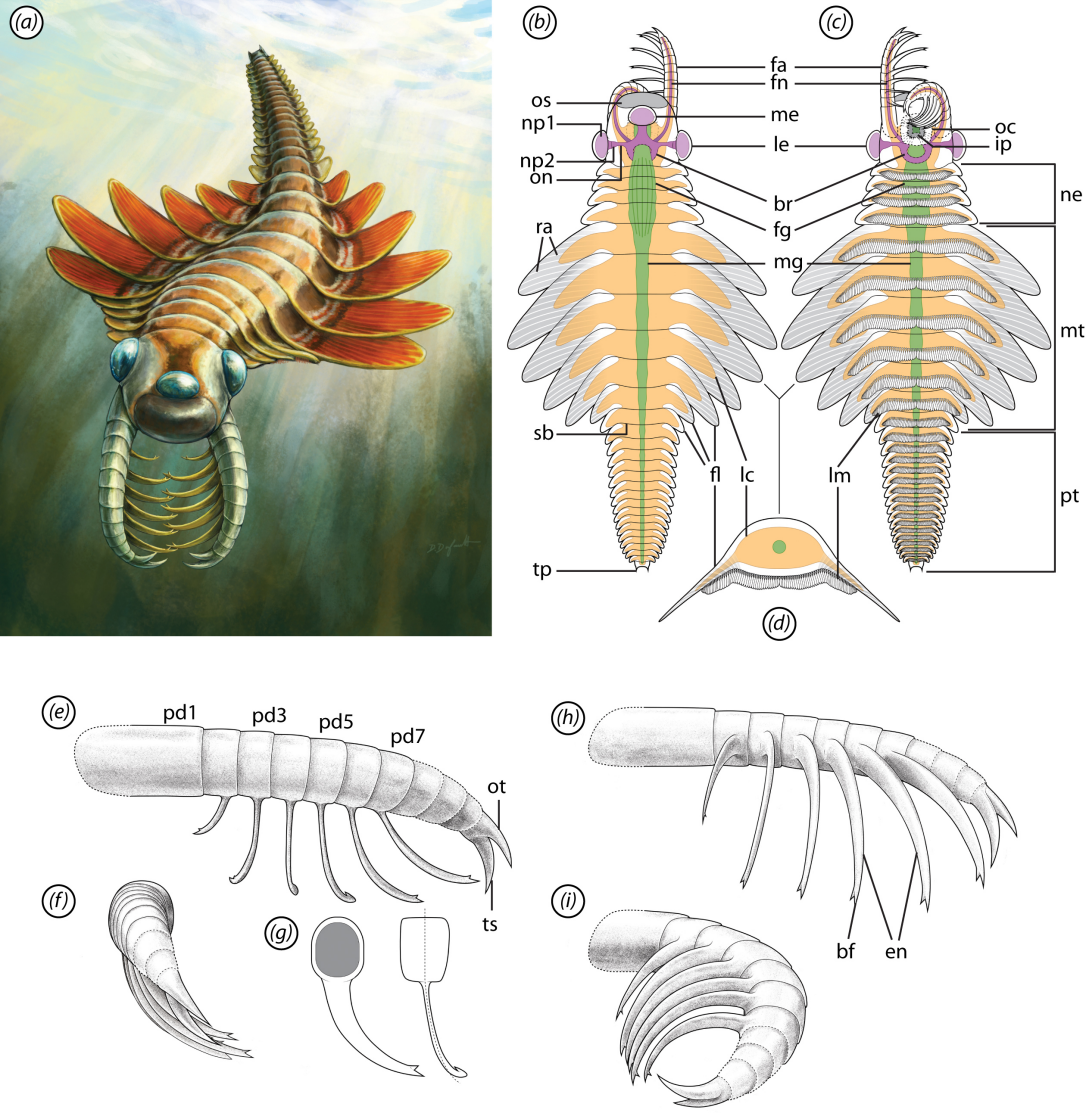
Morphological summary and artistic reconstruction. (*a*) Life reconstruction; (*b*) whole body, dorsal view; (*c*) ventral view; (*d*) cross-section through mesotrunk; (*e*) appendage, lateral view; (*f*) appendage, frontal view; (*g*) podomere 5 from lateral and frontal views; (*h*) appendage, ventral view, extended; (*i*) appendage, ventral view, retracted. Abbreviations: bf, bifurcated tip of endite; br, brain; en, endite; fa, frontal appendage; fg, foregut; fl, flap; fn, frontal appendage nerve/circulatory lacuna; ip, putative inner plates of oral cone; lc, lacuna of circulatory system; le, lateral eye; lm, band of lamellae; me, median eye; mg, midgut; mo, mouth; mt, mesotrunk; ne, neck; np#, lateral eye neuropil #; oc, oral cone; on, optic nerve; os, preocular sclerite; ot, outer spine of appendage; p, phosphatized element; pd#, podomere #; pt, posterotrunk; ra, flap ray; sb, segmental boundary; tp, terminal process; ts, terminal spine of appendage. Elements shown in dashed lines (appendage podomeres, oral plates, ring-shaped brain) are uncertain and have been inferred. Artwork by Danielle Dufault © Royal Ontario Museum.

See figure 7 for a summary of morphological observations.

## 4. Results

### 4.1. Phylogenetic results

**Figure 8.**
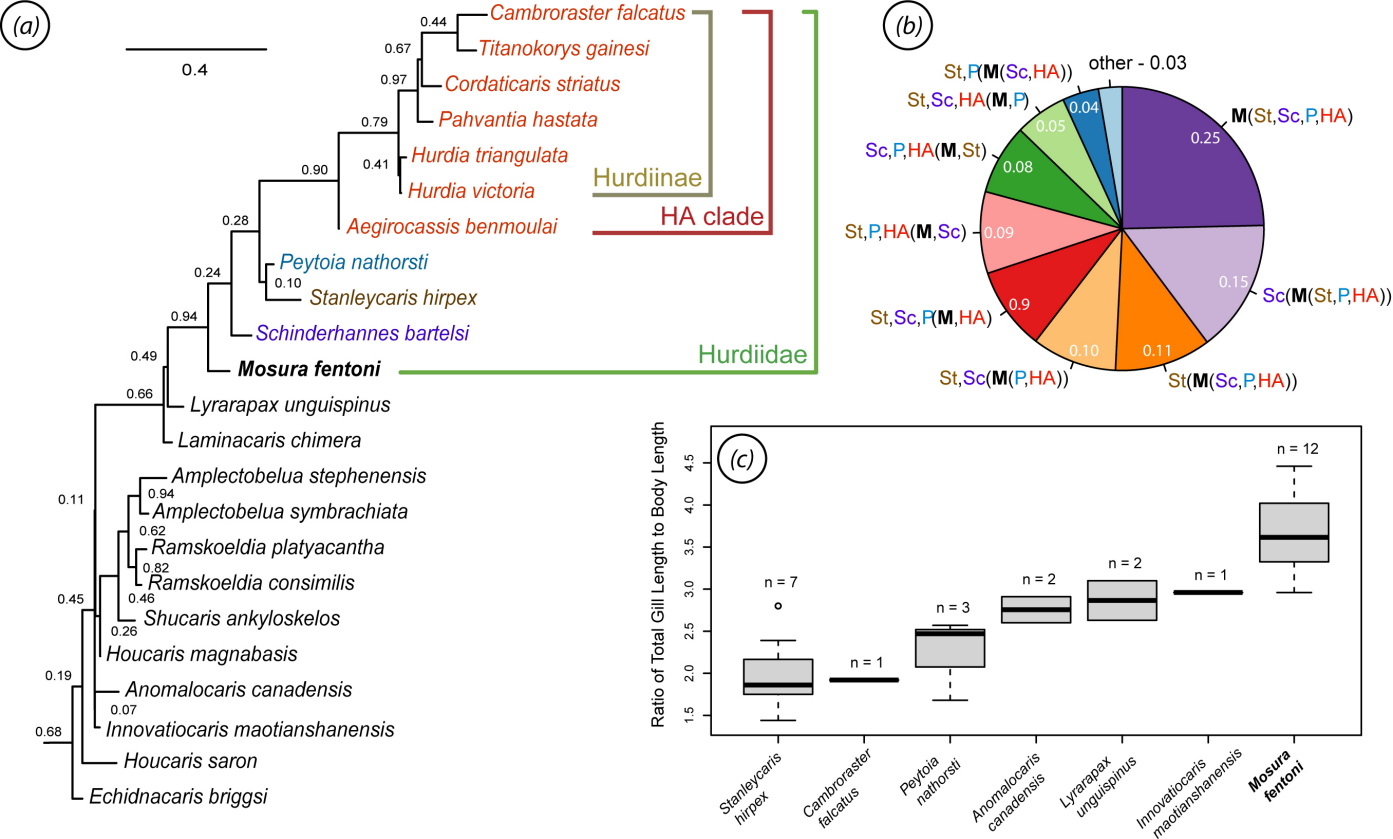
Phylogenetic affinities and gill measurements. (*a*) Pruned MCC tree with posterior probabilities shown at nodes (see electronic supplementary material, figure S8, for full trees); (*b*) pie chart representing the proportion of posterior trees with the indicated topology in parenthetical notation, using abbreviated taxon names: HA, Hurdiinae + Aegirocassisinae clade; M, *Mosura*; P, *Peytoia*; Sc, *Schinderhannes*; St, *Stanleycaris*; (*c*) boxplot showing relative gill lengths for several radiodont taxa.

*Mosura* exhibits a combination of traits seen in hurdiids and the paraphyletic non-hurdiids. For example, the single row of six elongate, mesially curving endites on the frontal appendages is an unambiguous apomorphic configuration for Hurdiidae. The inner plates in the oral cone are unique to hurdiids, though not found in all species. Similarly, the tetraradial arrangement of the oral plates and absence of posterior auxiliary spines on the frontal appendage endites are typical of, though not unique to, hurdiids. On the other hand, the elongate, multisegmented body, short head, distinct, constricted neck region and small rounded preocular sclerite are traits more often seen in non-hurdiids such as *Anomalocaris* [34] or *Amplectobelua* [42], although these characteristics are also shared with the basal hurdiid *Stanleycaris* [41]. It is therefore logical that we recover *Mosura* as part of a paraphyletic basal grouping of hurdiids, alongside *Stanleycaris*, *Schinderhannes* and *Peytoia* (figure 8*a*; electronic supplementary material, figure S6). Hurdiidae is strongly supported, with 0.94 posterior probability. We also find good support (0.90) for a clade including *Aegirocassis*, *Hurdia*, *Cordaticaris*, *Pahvantia*, *Cambroraster* and *Titanokorys*, here termed the Hurdiinae + Aegirocassisinae or HA clade (see electronic supplementary material for a description of subfamily Hurdiinae).

Within the basal grade of hurdiids, there is uncertainty as to the placement of *Mosura*. In the MCC tree (figure 8*a*), *Mosura* is resolved as the sister taxon to all other hurdiids while in the majority rule consensus (electronic supplementary material, figure S6), *Mosura* is found in a polytomy with *Stanleycaris, Schinderhannes*, *Peytoia* and the HA clade. Further investigation of the posterior tree set using monophyly testing indicates that the most likely placements for *Mosura* are as (i) sister to all other hurdiids (0.25); (ii ) sister to *Stanleycaris + Peytoia *+ HA (0.15); or (iii) sister group to *Schinderhannes + Peytoia *+ HA (0.11) with several other possible minority configurations obtaining values of less than 0.10 posterior probability (figure 8*b*). This uncertainty is likely due to the fact that *Mosura* is quite autapomorphic and shares relatively few characters with other hurdiids. Further conflict likely comes from the combination of plesiomorphic (straight endites, short head) and derived (loss of distinguishable neck region, low number of body segments) states in the poorly known genus *Schinderhannes*. Indeed, several of the minority topologies vary only in their placement of *Schinderhannes*. When *Schinderhannes* is removed, *Mosura* plots as sister to all other hurdiids with a probability of 0.44 while the probability that *Stanleycaris* is sister to all other hurdiids (including *Mosura*) is 0.39.

The finding of *Lyrarapax* and *Laminacaris* as nested sister taxa to Hurdiidae in the MCC tree is also noteworthy. A similar topology was previously recovered [36], but the inclusion of at least *Lyrarapax* within Amplectobeluidae has been more generally supported [37,41,61,64,65]. This result is likely strongly influenced by the coding of *Lyrarapax*, which has a tetraradial oral cone like hurdiids [55], but lacks evidence for gnathobase-like structures as found in *Amplectobelua*, *Ramskoeldia* and *Shucaris* [39,65]. For now, knowledge of the distribution of gnathobase-like structures and oral cone morphologies remains patchy due to the incompleteness of many radiodont taxa, making this topology unstable, as indicated by the poorer resolution in the majority rule consensus tree.

### 4.2. Gill morphometrics

*Mosura* has the highest ratio of total gill length to body length of any of the taxa considered, averaging about 3.7 (figure 8*c*). This is presumably heavily influenced by the fact that it has a greater number of segments than other taxa. *Cambroraster*, *Peytoia* and *Stanleycaris* have the lowest ratios (typically less than 2.5), while *Anomalocaris*, *Lyrarapax* and *Innovatiocaris* are somewhat intermediate. There is considerable variance within taxa, presumably due to individual and taphonomic variation. Small sample sizes for most species make statistical testing infeasible, but the distribution of *Mosura* stands out as essentially non-overlapping with other species, with only marginal overlap at the lowest extreme with the highest values for *Lyrarapax* and *Innovatiocaris*.

## 5. Discussion

### 5.1. Lacunar systems in early arthropods

Exceptional preservation of the internal organs of *Mosura* complements what is known in other radiodonts, as well as adding new insights. Details of the nervous and digestive systems correspond well with what can be observed in some of the best-known radiodonts like *Stanleycaris* [41] and *Lyrarapax* [37,55]. The haemolymph lacunar system is particularly well-preserved and warrants special discussion.

In euarthropods and onychophorans, the lacunar system is one of two main components of the circulatory system, the other being the vascular system [66]. The vascular system typically consists of a heart and arteries supplying haemolymph to various parts of the body. The lacunar system consists of a series of expansive haemolymph sinuses, or lacunae, surrounding the vascular system and other internal organ systems. Haemolymph passes from the vascular system into the lacunar system where it is eventually channelled back to the heart. In *Mosura*, we interpret the large, reflective internal stains with triangular extensions into the flaps as the traces of the lacunar system.

Similar dark, reflective stains with aluminosilicate or phosphatic composition within the bodies of Cambrian arthropods (sometimes neutrally referred to as ‘tonguelettes’ [67]) have a history of controversial interpretation. These structures have been variously considered to be components of the circulatory system [12,46,68,69], gut diverticulae [12,68,70], externally developed lobopodous appendages [71,72] or ganglia of the ventral nerve cord [73–77]. We consider that several recent advances have made the case that a circulatory, and specifically lacunar, interpretation of the tonguelettes is by far the most plausible. First, new fossils have confirmed that the tonguelettes are internal structures, contained within the body and appendages, and are anatomically separate from the gut [12,14,41,78,79]. This is reinforced by *Mosura* material, in which they are associated with swimming flaps and preserved distinct from the gut (figures 4*a*–*e* and 6*c,d*; electronic supplementary material, figure S2a–c,f,g). The presence of tonguelettes inside both flaps [41,78] and arthrodized limbs [14] demonstrates that they are not uniquely associated with lobopods. Second, new approaches have provided improved insight into lacunar anatomy in extant panarthropods [80–82]. Detailed morphology of tonguelettes is much more compellingly compared with haemolymph lacunae than with the nervous system or digestive diverticulae, and in some cases, tonguelettes may co-occur with the last two, violating the homological test of congruence [67]. Third, decay experiments have shown that tonguelette-like structures can be replicated through microbial pseudomorphing of haemolymph lacunae, providing a plausible taphonomic pathway [83]. The implication of this interpretation is that the lacunar system is widely preserved in Cambrian euarthropods, radiodonts and related taxa like *Opabinia*. Interpretations of other systems, such as the nervous system, must therefore take into account the likelihood of the preservation of surrounding lacunar traces and the possibility of confounding anatomically overlapping structures [67,84].

**Figure 9.**
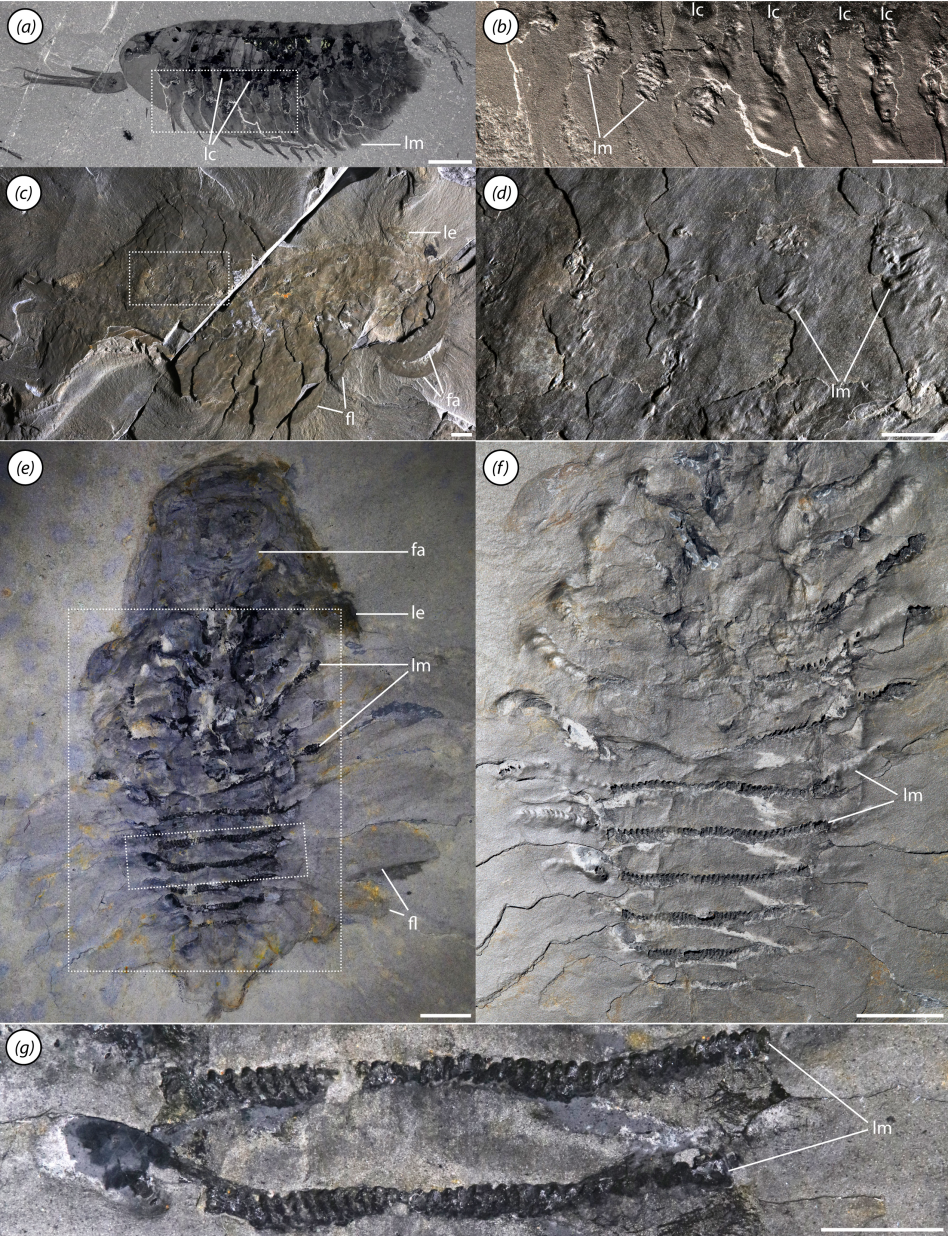
Comparison of phosphatized gill structures in radiodonts and megacheirans. (*a,b*) The megacheiran *Yawunik kootenayi*, ROMIP 68652, (*a*) overall specimen, (*b*) close-up of lamellate structures; (*c,d*) the radiodont *Anomalocaris canadensis*, ROMIP 51214, (*c*) overall specimen, (*d*) close-up of lamellate structures; (*e–g*) the radiodont *Peytoia nathorsti*, USNM 274141, (*e*) overall specimen, (*f*) close-up of trunk region, (*g*) close-up of chain-like structures representing the bases of bands of gill lamellae. Scale bars, (*a,c,e,f*) 10 mm; (*b,d,g*) 5 mm. Abbreviations: fa, frontal appendage; fl, flap; lc, lacuna of circulatory system; le, lateral eye; lm, band of lamellae.

In *Mosura* (as in *Stanleycaris* [41]), the extent of the trunk lacunae mirrors that of the bands of gill lamellae, implying a close anatomical association. Several other Cambrian arthropods also show a similarly close spatial relationship between lacunae and appendage rami (typically exopods) with lamellate gills [14,85] (figure 9). This organization is comparable to that seen in extant euarthropods [81,82] and onychophorans [80], in which lacunae extend deep into the limbs and, where present, into specialized respiratory outgrowths. This enables the exchange of gases between gills, haemolymph and locomotory muscles.

This close anatomical association may also help to explain the occasional selective phosphatization of lacunae, gills and putative patches of adjacent muscle tissue in a variety of Cambrian panarthropods. Lacunae likely acted as conduits within the decaying carcass [83], enabling microbes and phosphate to move from the gut after it ruptured to other areas, such as gills and adjacent locomotory muscles, providing the necessary ingredients for early phosphatization in *Mosura* and other arthropods in Burgess Shale-type deposits (figure 9) [33,34,37,41,86,87]. We speculate that gills could be particularly prone to phosphatization due to their high surface area, thin cuticle and presumably rapid decay creating a conducive microenvironment [41,88]. Interestingly, unlike many Burgess Shale euarthropods [89], the guts of *Mosura* and other radiodonts tend to be weakly phosphatized, if at all. One possible explanation is that the gut rupture occurred rapidly, disrupting the intra-gut microenvironment before significant phosphatization could take place. The midgut is often incompletely preserved in *Mosura*, suggesting that rupture may have already occurred. Some specimens of *Mosura*, particularly from Marble Canyon and Tokumm Creek, exhibit more extreme overgrowth of large parts of the body with calcium phosphate. This pattern is unique at the Burgess Shale and its taphonomic explanation remains to be explored.

### 5.2. A small, active, nektonic hunter

*Mosura* adds to a growing list of radiodont species in which a median eye has been described [41], but the functional role of this structure has not been discussed. It is not yet clear whether this median eye had a compound or single-lens structure, although the latter is favoured by comparison with putatively homologous median eyes in extant euarthropods. The large size and hemiellipsoidal shape of the radiodont median eye are unusual for arthropod single-lens eyes, but a possible functional analogy can be drawn with the central member of the triplet of median eyes found in dragonflies. Here, the hemiellipsoidal shape of the large median eye has been suggested to be optimized for the detection of horizontal structures such as the horizon line, which aids in maintaining orientation during rapid aerial manoeuvres [90]. This is interesting given other similarities between dragonflies and radiodonts. First, many radiodonts are hypothesized to have been fast-swimming hunters and exhibit morphologies consistent with considerable manoeuvrability in the water column [44]. Second, the lateral compound eyes of radiodonts have a large number of lenses, in some cases rivalling the number seen in dragonflies, which would have provided excellent visual acuity when pursuing mobile prey [41,91,92]. Like dragonflies, the lateral eyes of radiodonts would have been the primary sensory organs used in perceiving details of the surrounding environment such as prey [91], and we postulate that the median eye may have functioned to enable the maintenance of orientation during fast manoeuvres.

Additional evidence for an active, macrophagous lifestyle comes from the appendage morphology. The presence of six elongate, mesially curving, bladelike endites is a hurdiid apomorphy [36]; however, *Mosura* is distinct in lacking the pectinate array of auxiliary spines on its endites that otherwise characterize members of this clade [61]. Indeed, the presence of multiple elongate endites in radiodonts is typically associated with suspension feeding or sediment sifting [36,93,94], but the absence of spines or setae to form a feeding ‘mesh’ precludes microphagy in *Mosura*. Rather, functional morphology is more consistent with the endites serving as hooks to capture larger prey. In most specimens, the appendages are preserved tightly tucked below the head, but one (figure 2*a*–*d*) shows that they were also capable of flexing forward such that the endites project anteriad. In the absence of a confident modern analogue, we propose that *Mosura* could have hooked prey with the endites, then rotated its appendages backward to bring it towards the mouth for ingestion.

The overall body morphology of *Mosura*, with a relatively short head, small preocular sclerite, anteriorly positioned eyes, long, multisegmented body and wide swimming flaps are suggestive of a nektonic lifestyle, more comparable to non-hurdiids like *Anomalocaris* than nektobenthic hurdiids like *Cambroraster*. Among hurdiids, only *Stanleycaris* has a similar gross body morphology, and this genus is also suggested to have had strong swimming capability [41,64]. It is likely that the large mesotrunk flaps of *Mosura* were the primary means of propulsion, which presumably functioned similarly to flaps in other radiodonts [95]. That said, the unique morphology of the posterotrunk and the absence of posterior blades or filaments, found in some form in most other radiodont species [44], likely had functional implications for swimming mechanics and manoeuvrability which remain to be evaluated.

*Mosura* shared its environment with *Cambroraster* and *Titanokorys* at the Marble Canyon and Tokumm Creek localities and with *Anomalocaris*, *Hurdia* and *Peytoia* in the Raymond Quarry. Although the details of niche partitioning in Cambrian ecosystems remain challenging to infer, body size has been hypothesized to be a potentially important factor in partitioning food resources [96]. In addition to functionally pertinent differences in morphology, the maximum body size of *Mosura* appears to be notably smaller than sympatric, macrophagous radiodont species, which suggests specialization on a smaller prey size fraction.

### 5.3. A specialized respiratory tagma in an early arthropod

The most striking features of *Mosura* are the high number of trunk segments and the marked differentiation of segment and flap size between the meso- and posterotrunk. The sharp boundary between trunk segment batches contrasts with the typical radiodont condition in which posterior flaps exhibit a continuously decreasing size gradient [97]. The lateral flaps on the posterotrunk appear to be too small to have played a significant role in locomotion. In contrast, the bands of lamellae, which presumably functioned as gills [56] are well developed on all trunk segments. Although gills are not located exclusively on the posterotrunk, this posterior tagma does comprise the majority of gill-bearing segments. Functional specialization of the posterotrunk for respiration thus appears to be the most plausible hypothesis to explain this morphology. Considering the evidence for morphological and functional differentiation between the mesotrunk and posterotrunk, we consider these regions to qualify as distinct tagmata.

Several arthropod groups have evolved a posterior tagma with extreme modification and reduction of appendages, associated with respiratory adaptations. In isopods, five posterior segments comprising a tagma known as the pleon are frequently reduced in size, and their appendages (pleopods) are modified as a series of overlapping, flattened structures [98]. The pleopods function primarily as respiratory and osmoregulatory organs. A similar situation can be found in xiphosurans, in which most appendages of the posterior opisthosoma are modified into lamellate book gills that primarily function for respiration [99]. Hexapods also exhibit extreme reduction and modification of abdominal appendages, remnants of which form the respiratory tracheae [100,101] and lobate tracheal gills in some aquatic larvae [102]. Perhaps the most compelling morphological comparison is with the opisthothorax of some redlichiid trilobites. Similar to *Mosura*, this posterior tagma consists of a large number of segments (up to 97), which are reduced in size and tightly spaced relative to the segments of the prothorax [103]. Unfortunately, the trunk appendage morphology of trilobites with such a bipartite trunk remains unknown and the function of the opisthothorax remains unclear. Regardless, the cases above illustrate multiple convergent origins of a posterior tagma characterized by segment and appendage reduction, primarily specialized for respiratory functionality. If this functional interpretation similarly holds for the posterotrunk of *Mosura*, this is the first documented case of such a tagma outside of Euarthropoda.

The presence of a large, specialized respiratory tagma may suggest that *Mosura* had a physiological requirement for elevated respiratory performance relative to other radiodonts. Although we are not able to precisely quantify radiodont gill surface area or body volume, our measures of total gill length and body length provide a crude estimation. *Mosura* exhibits the highest relative gill length among radiodonts, despite being among the smallest known in terms of adult body size, a surprising finding given that gill area often exhibits higher allometric scaling coefficients as adult body size increases [104].

There are several possible explanations for the increased gill surface area in *Mosura*. First, alongside body size reduction, increasing gill area is considered to be an adaptation to low-oxygen environments in some extant crustaceans [105]. Such low-oxygen conditions were widespread in Cambrian outer shelf environments and have even been implicated in Burgess Shale-type preservation [106,107]. The hypothesis that *Mosura* was adapted to relatively low-oxygen conditions is seemingly contradicted by the fact that it co-occurs with larger radiodonts that have lower relative gill lengths, such as *Anomalocaris* and *Hurdia* in the Raymond Quarry or *Cambroraster* and *Titanokorys* in the Marble Canyon and Tokumm Creek sites. However, this observation can be reconciled if these animals inhabited distinct life environments and were brought together through transport into a common burial assemblage [108]. In this context, it is noteworthy that all known *Mosura* specimens consist of articulated body remains, whereas co-occurring *Anomalocaris*, *Hurdia*, *Cambroraster* and *Titanokorys* are primarily represented by disarticulated elements and semi-articulated exuviae [34–36,96]. It is therefore plausible that *Mosura* inhabited distinct, oxygen-stressed habitats, proximal to the site of burial.

If not an environmental adaptation, the elevated respiratory surface area in *Mosura* could alternatively have been an adaptation to distinct behavioural traits. In extant crustaceans, a more active lifestyle or greater reproductive output necessitates greater oxygen consumption and larger gills [104]. Similarly, the difference in lifestyle between nektonic raptorial predators like *Anomalocaris* and nektobenthic sediment-sifting hurdiids like *Cambroraster* [36,109] appears to be a potential explanation for the observed differences in trunk morphology, and specifically relative gill length, in these taxa. A similar difference in lifestyle could also have driven the evolution of the specialized trunk of *Mosura*, though its precise nature is less clear in this case. At the very least, this would seem consistent with the inferred active, macrophagous, predatory lifestyle indicated by other aspects of morphology discussed previously. At present, it therefore remains uncertain whether environmental, ecological or a combination of factors contributed to selecting for enhanced respiratory performance and the consequent evolution of the multisegmented posterotrunk in *Mosura*.

Functional specialization of serially repeated structures like segments enables release from trade-offs and individual optimization of tagmata. This has clearly been a major factor in the diversification of arthropods [2]. For example, certain functions like sensation and feeding are nearly always localized to anterior segments, which is intuitively sensible in animals that move forward and have anteriorly located mouths [5]. Could a similar mechanism have driven the repeated evolution of posterior respiratory tagmata in *Mosura* and diverse arthropod groups? We consider it unlikely that selection directly favoured the partitioning of respiratory functionality to posterior appendages. First, there is no reason to expect that respiratory performance would be generally enhanced in posterior segments, or in any other particular location. Second, there exist several counter cases indicating alternative optimization strategies, for example, the specialization of intermediate appendages of the thorax (pereon) for respiration in eucarid crustaceans [7]. It would seem more likely that posterior respiratory tagmata evolve as a side consequence of the partitioning of other functional roles such as locomotion towards the anterior. In this sense, while functional specialization itself was likely adaptive, the evolution of a respiratory tagma at the posterior of the body could be considered a spandrel, *sensu* [110]. A theoretical morphology perspective, considering the impact of flap number, relative size, and position on hydrodynamic performance, could be a fruitful approach to further explore the hypothesis of functional trade-offs between respiratory area and swimming performance in radiodonts.

*Mosura* is one of only three radiodonts with known juvenile ontogenetic stages [41,55], which provides an opportunity to consider the developmental origin of its distinctive trunk tagmosis. Smaller specimens are distinguished by a lower number of segments in the posterotrunk, and possibly also in the mesotrunk. An increase in total segment number during ontogeny, hemianamorphosis, is consistent with previous observations in *Stanleycaris* [63]. If the increase in mesotrunk segment number is genuine, and not due to the limits of visual resolution imposed by preservation in small specimens, this also implies that some segments from the posterotrunk would have differentiated during ontogeny and become incorporated into the mesotrunk. Such a growth mode is uncommon, though observed in a few arthropod groups [111]. However, the hypothesis of biphasic growth remains uncertain until additional specimens are recovered.

The trunk tagmosis in *Mosura* represents a considerable deviation from that seen in other radiodonts, indicating heretofore unappreciated variability in this clade. Although the disparity of tagmosis and the extent of segment differentiation between tagmata in radiodonts still pales in comparison to that seen in many euarthropod clades, it is notably greater than that of extant onychophorans and tardigrades and comparable to that of Palaeozoic lobopodians. Indeed, *Mosura* shares a posterior respiratory tagma with some of the most successful and long-lived euarthropod lineages. This adds to a growing number of cases of convergent traits observed in radiodonts and euarthropods [22,36,55,64,93], demonstrating the capacity to adapt to similar environmental challenges in similar ways. The recognition that key characteristics associated with arthropod evolvability were already manifest in Cambrian radiodonts serves to further emphasize the importance of evolutionary dynamics in the Cambrian in shaping the longer-term structure of arthropod disparity. This would argue against the idea that radiodont extinction was driven by lower evolvability of tagmosis than contemporaneous euarthropods. Rather, it appears to better fit a broader pattern of high initial variability, giving rise to multiple parallel increases in panarthropod tagmosis, followed by non-selective extinction and canalization in surviving clades.

## Data Availability

All data and code associated with this paper are provided as electronic supplementary material. Requests for images should be made to J.-B.C. at jcaron@rom.on.ca. Supplementary material is available online [112].
